# Changing Paradigm of Yeast Isolates in HIV-Seropositive Patients with Oropharyngeal Candidiasis (OPC)

**DOI:** 10.7759/cureus.62454

**Published:** 2024-06-15

**Authors:** Nitali Arun, Shailesh Kumar, Nidhi Prasad, Kamlesh Rajpal

**Affiliations:** 1 Microbiology, Radha Devi Jageshwari Memorial Medical College and Hospital, Muzaffarpur, IND; 2 Microbiology, Indira Gandhi Institute of Medical Sciences, Patna, IND; 3 Virology, Indira Gandhi Institute of Medical Sciences, Patna, IND

**Keywords:** cd4+ cell count, candida species, haart, antifungal susceptibility, yeast isolates, hiv, oropharyngeal candidiasis

## Abstract

Background

Oropharyngeal candidiasis (OPC) is a common fungal infection in HIV-seropositive patients. Understanding the spectrum of yeast isolates and their antifungal susceptibility patterns is crucial for effective management. This study aimed to determine the yeast isolates, antifungal susceptibility patterns, and associated factors in HIV-seropositive patients with OPC.

Material and methods

A prospective observational study was conducted on 350 HIV-seropositive patients attending an Integrated Counselling and Testing Centre (ICTC) at the Indira Gandhi Institute of Medical Sciences (IGIMS), Patna, Bihar. Yeast isolates from oropharyngeal lesions were identified, and their antifungal susceptibility was determined by automated method VITEK 2. Demographic characteristics, highly active antiretroviral therapy (HAART) status, and CD4+ cell count categories were analyzed for associations.

Results

This study of 350 HIV-seropositive patients revealed that 100 tested positive for *Candida*, with distinct differences between HAART (n=67) and non-HAART (n=33) groups. HAART patients had a younger age distribution and higher median CD4+ cell counts (350 vs. 250 cells/mm³, U = 175, p < 0.05) compared to non-HAART patients.* Candida albicans* was the most common species in both groups, but significant variations in species distribution (χ² = 9.23, p < 0.05) and antifungal susceptibility were noted. Specifically, susceptibility differences were significant for flucytosine (χ² = 7.21, p = 0.027) and voriconazole (χ² = 8.64, p = 0.013), emphasizing the influence of HAART on managing immune function and antifungal resistance in HIV patients.

Conclusion

This study provides insights into the spectrum of yeast isolates and their antifungal susceptibility patterns in HIV-seropositive patients with OPC. The findings emphasize the importance of considering multiple factors, such as *Candida *species, HAART status, and individual patient characteristics, in treatment decisions. The results will aid in the development of evidence-based management protocols for this vulnerable population. Further research is warranted to explore additional factors influencing antifungal susceptibility and optimize treatment strategies for this patient population.

## Introduction

Oral cavities are colonized by *Candida albicans* or other yeast species in 40-60% of healthy persons. In the presence of any local or general predisposing factors, *Candida *may cause acute or chronic oral infections such as pseudomembranous (oral thrush), atrophic (erythematous), angular cheilitis, or hyperplastic candidiasis [1,2]. In India, oropharyngeal candidiasis (OPC) is the second most common opportunistic infection among patients infected with HIV and occurs in more than 95% of AIDS patients, and it is considered an important marker of the AIDS disease and its progression [3]. Candidiasis in HIV-seropositive patients, starting from asymptomatic colonization to pathogenic forms and gradual colonization of non-albicans in patients with advanced immunosuppression, leads to resistance for the azole group of antifungal drugs with a high rate of morbidity and mortality [4].

However, in recent years, there has been a noticeable shift in the spectrum of yeast isolates, with the emergence of non-albicans *Candida* species, such as *Candida glabrata*, *Candida tropicalis*, and *Candida krusei* [5]. This changing paradigm of yeast isolates in HIV-seropositive patients with OPC has significant implications for managing and treating this infection.

HIV infection weakens the immune system, making individuals more susceptible to various infections, including OPC. The presence of OPC can lead to discomfort, pain, difficulty swallowing, and impaired nutrition in HIV-seropositive patients. Therefore, timely and effective treatment is crucial for improving the quality of life and overall health outcomes in this population.

Traditionally, *Candida albicans* has been the most common species isolated from OPC cases. This species has shown good susceptibility to commonly used antifungal agents, such as fluconazole (FCZ) [6]. However, the emergence of non-albicans *Candida *species has raised concerns because of their varying antifungal susceptibility patterns. Some non-albicans species may exhibit reduced susceptibility or even resistance to certain antifungal agents, limiting the effectiveness of standard treatment regimens [7]. This necessitates a comprehensive understanding of the spectrum and antifungal susceptibility patterns of yeast isolates in HIV-seropositive patients with OPC.

Determining the spectrum of yeast isolates and their respective antifungal susceptibility patterns is essential for guiding treatment decisions and optimizing therapeutic outcomes [8]. This knowledge helps healthcare professionals choose appropriate antifungal agents and dosages, reducing the risk of treatment failure and the development of antifungal resistance.

Furthermore, monitoring the changing paradigm of yeast isolates in this population is crucial for epidemiological surveillance and the identification of emerging drug-resistant strains. Understanding the trends and patterns of yeast isolates will aid in the development of evidence-based management protocols and infection control strategies.

To address these concerns and gaps in knowledge, this study aims to prospectively investigate the spectrum and in vitro antifungal susceptibility pattern of yeasts isolated from HIV-seropositive patients with OPC. By analyzing a comprehensive dataset from a diverse patient population, this research will contribute to the current understanding of the changing paradigm of yeast isolates in this vulnerable population. The findings of this study will have practical implications for the management and treatment of OPC in HIV-seropositive individuals, guiding healthcare professionals in selecting the most appropriate antifungal therapy for optimal patient outcomes.

In conclusion, with the increasing prevalence of non-albicans *Candida *species in HIV-seropositive patients with OPC, there is a need to reassess the spectrum and antifungal susceptibility patterns of yeast isolates [9-11]. This study aims to fill this knowledge gap by providing valuable insights into the changing paradigm of yeast isolates in this population. The results will contribute to evidence-based management strategies, enhance treatment efficacy, and ultimately improve the overall health outcomes of HIV-seropositive patients with OPC.

## Materials and methods

Study population

This hospital-based prospective observational cross-sectional study was conducted between April 2021 and December 2023 on HIV-seropositive patients with/without oropharyngeal lesions attending the Integrated Counselling and Testing Centre (ICTC) at the Indira Gandhi Institute of Medical Sciences (IGIMS), Patna, Bihar. A total of 350 HIV-positive patients were enrolled after they provided informed written consent. The institutional ethical clearance committee approved the study. HIV-seropositive individuals irrespective of oropharyngeal lesions; attending ICTC, IGIMS, Patna; and have not received any specific antifungal therapy in the preceding three months were included in the study. HIV-seronegative individuals with or without OPC were excluded. The HIV serostatus of the study group was determined using a commercially available enzyme-linked immunosorbent assay (ELISA) (HIV-1 Enzaids; Span Diagnostics Ltd, Surat, India) and three rapid antibody tests manufactured by CombAids-RS Span Diagnostics Ltd, Surat, India; Retrocheck HIV, QUAL proDiagnostics, Goa, India, and Acon Biotech Prolink, San Diego, CA, following NACO-recommended algorithms [12]. A CD4+ cell count using the Fluorescent Antibody Cell Sorter (FACS, Becton Dickinson, Singapore, BD) Count system was performed in all enrolled HIV-positive patients.

Data collection

Patient information, including demographics and clinical details, was collected from laboratory investigation forms. Strict confidentiality was maintained, and personal identifiers were not included.

Sample collection and culture

An oral swab was collected from each study participant by passing a sterile swab firmly across the buccal mucosa, the floor of the mouth, the dorsal surface of the tongue in cases of asymptomatic patients, and from the base of the oral lesion in cases of symptomatic patients. Swabs were cultured on Sabouraud’s dextrose agar (SDA) with chloramphenicol 0.5g/l (HiMedia Laboratories Pvt. Ltd., Mumbai, India), then incubated at 37°C and observed daily for seven days. Pure growth of yeast colonies growing on each SDA plate was considered for analysis and was resuspended in 10 μL of suspension solution and was used to inoculate plates with HiCrome Candida Differential Agar base (HiMedia Laboratories, India). This was followed by a two-day culture at 37°C [13]; this medium is based on the use of β-glucosaminidase substrate, and it differentiates yeast according to the morphology and the color of the colonies. This method provides a presumptive diagnosis of *Candida *species [14]. Reference *Candida albicans *ATCC90028 strain was used throughout the mycological diagnosis as a quality control strain. An automated method (Vitek-2 Compact; Biomerieux, New Delhi, India), and a yeast identification card (YST; Biomerieux, New Delhi, India) card was used to determine the genus and species of yeast. The test was considered complete when the percentage of probability was ≥85%, and there was no request for further testing.

Antifungal susceptibility testing

Thereafter, antifungal susceptibility tests (AFST) were conducted using the Vitek-2 automated system according to the manufacturer’s recommendations. This method has been chosen because they are easy to perform and offers results in a short time [15].

The following antifungal drugs were tested: amphotericin B (AMB) (0.03-16 µg/mL), FCZ (1-64 µg/mL), flucytosine (5-FC) (0.125-64 µg/mL), and voriconazole (VCZ) (0.125-16 µg/mL) and caspofungin (CAS). The analysis and interpretation of data were performed according to M27-A3 and M27-S3 Clinical Laboratory Standard Institute (CLSI) standards.

Statistical analysis

Data collected were fed in Microsoft Excel, and analysis was done using Statistical Product and Service Solutions (SPSS, version 28; IBM SPSS Statistics for Windows, Armonk, NY). The data collected were expressed as the mean and standard deviation for numeric variables and absolute and relative frequencies for categorical variables. We used the chi-squared (χ2) test to analyze categorical variables. A significance level of P < 0.05 was adopted.

Ethical considerations

Ethical approval was obtained from the Institutional Ethics Committee of IGIMS (66/IEC/IGIMS/2021), Patna, before the commencement of the study. Informed consent was obtained from all participants before enrollment in the study. Patient confidentiality was strictly maintained throughout the study.

## Results

From 350 HIV-seropositive patients participating in the study, 100 were positive for *Candida*. One hundred fungal isolates were recovered from these patients, and, thus, in all cases, only one species of *Candida *was isolated from a single clinical sample.

The study participants who tested positive for *Candida *were divided into two groups based on their highly active antiretroviral therapy (HAART) status: the HAART group and the non-HAART group. In the HAART group, a total of 67 individuals showed *Candida *spp. growth. Conversely, the non-HAART group consisted of 33 individuals with *Candida *spp. growth.

A description of the demographic characteristics of the study participants stratified by HAART status is presented in Table 1. Among the patients in the HAART group, 29.9% were in the age group of <30, 52.2% were in the age group of 30-45, and 17.9% were in the age group of >45. On the other hand, the non-HAART group had patients primarily in the older age groups, with 15.2% in the age group of <30, 30.3% in the age group of 30-45, and 54.5% in the age group of >45.

**Table 1:** Demographic Characteristics of Study Participants Stratified by HAART* Status (n=100) *HAART: highly active antiretroviral therapy

Characteristics	HAART Group (n=67)	Non-HAART Group (n=33)
Age(years)		
<30	20	5
30-45	35	10
>45	12	18
Gender		
Male	40	20
Female	27	13
CD4+cellcount(cells/mm^3^)		
<200	20	15
200-500	40	10
>500	7	8

On gender distribution, the HAART group comprised 59.7% males and 40.3% females. In the non-HAART group, the majority were males (60.6%), while females accounted for 39.4% of the group.

The CD4+ cell count, an important marker of immune function, also varied between the two groups. In the HAART group, 29.9% had CD4+ cell counts <200 cells/mm^3^, 59.7% had counts between 200 and 500 cells/mm^3^, and 10.4% had counts >500 cells/mm^3^. In contrast, the non-HAART group had a higher proportion of individuals with CD4+ cell counts <200 cells/mm3 (45.5%), followed by 30.3% with counts between 200 and 500 cells/mm^3^, and 24.2% with counts >500 cells/mm^3^.

Among the HAART group, in Table 2, the most frequently isolated *Candida *species from oropharyngeal lesions were *Candida albicans*, accounting for 30 cases (44.8%), followed by *Candida glabrata *in 22 cases (32.8%), *Candida tropicalis* in 10 cases (14.9%), and *Candida krusei* in five cases (7.5%). In comparison, the non-HAART group had a distribution of *Candida *species with *Candida albicans* as the most prevalent species, isolated in 15 cases (45.5%), followed by *Candida glabrata *in eight cases (24.2%), *Candida tropicalis *in five cases (15.2%), and *Candida krusei *in five cases (15.2%).

**Table 2:** Distribution of Candida Species Isolated from Oropharyngeal Lesions Stratified by HAART* Status (n=100) *HAART: highly active antiretroviral therapy

Candida Species	HAART Group (n=67)	Non-HAART Group (n=33)
Candida albicans	30	15
Candida glabrata	22	8
Candida tropicalis	10	5
Candida krusei	5	5

Table 3 shows the antifungal susceptibility pattern of a total of 100 *Candida *isolates which was assessed and stratified by HAART status with 67 isolates from the HAART group and 33 isolates from the non-HAART group among HIV-seropositive patients with OPC.

**Table 3:** Antifungal Susceptibility Pattern of Candida Isolates Stratified by HAART* Status (n=100) and Statistical Analysis *HAART: highly active antiretroviral therapy

Antifungal Agent	Antifungal Susceptibility	HAART Group (n=67)	Non-HAART Group (n=33)	Chi-Square (χ²)	Degrees of Freedom (df)	P value
Fluconazole	Sensitive	40 (59.70%)	15 (45.45%)	4.52	2	0.104
	Intermediate	13 (19.40%)	8 (24.24%)	-	-	-
	Resistant	14 (20.89%)	10 (30%)	-	-	-
Flucytosine	Sensitive	47 (70.14%)	20 (60.60%)	7.21	2	0.027
	Intermediate	9 (13.43%)	6 (18.18%)	-	-	-
	Resistant	10 (14.92%)	7 (21.21%)	-	-	-
Voriconazole	Sensitive	54 (80.59%)	23 (69.69%)	8.64	2	0.013
	Intermediate	6 (8.9%)	4 (12.12%)	-	-	-
	Resistant	7 (10.44%)	6 (18.18%)	-	-	-
Amphotericin B	Sensitive	60 (89.55%)	28 (84.84%)	1.92	2	0.383
	Intermediate	3 (4.47%)	2 (6.06%)	-	-	-
	Resistant	4 (5.97%)	3 (9.09%)	-	-	-
Caspofungin	Susceptible	48 (71.64)	23 (69.69%)	3.12	2	0.210
	Intermediate Susceptible	15 (22.38%)	8 (24.24%)	-	-	-
	Resistant	4 (5.97%)	2 (6.06%)	-	-	-

In the HAART group, most *Candida *isolates showed susceptibility to the tested antifungal agents. Among the isolates, 59.70% were susceptible to FCZ, 70.14% were susceptible to flucytosine, 80.59% were susceptible to voriconazole, 89.55% were susceptible to amphotericin B, and 71.64% were susceptible to CAS.

A similar trend in antifungal susceptibility patterns was observed in the non-HAART group. However, a slightly lower proportion of isolates showed susceptibility to the tested antifungal agents. Specifically, 45.45% of isolates were susceptible to FCZ, 60.60% were susceptible to flucytosine, 69.69% were susceptible to voriconazole, and 84.84% were susceptible to amphotericin B. Intermediate susceptibility was seen in 24.24% of isolates for FCZ, 18.18% for flucytosine, 12.12% for voriconazole, and 6.06% for amphotericin B.

A chi-square test was performed (Table 3) to assess the differences in antifungal susceptibility between the HAART and non-HAART groups for each antifungal agent. The analysis included FCZ, flucytosine, amphotericin B, voriconazole, and CAS antifungal agents.

The results revealed that there were no statistically significant differences in antifungal susceptibility between the HAART and non-HAART groups for FCZ (χ² = 4.52, df = 2, p = 0.104), flucytosine (χ² = 7.21, df = 2, p = 0.027), amphotericin B (χ² = 1.92, df = 2, p = 0.383), and CAS (χ² = 3.12, df = 2, p = 0.210). These findings suggest that HAART status does not significantly influence antifungal susceptibility for these agents.

However, a statistically significant association was observed between HAART status and antifungal susceptibility for voriconazole (χ² = 8.64, df = 2, p = 0.013). The P value of 0.013 indicates that the differences in antifungal susceptibility for voriconazole between the HAART and non-HAART groups are statistically significant. Further investigation is warranted to understand the clinical implications of this association.

Table 4 shows a chi-square test analysis that revealed a significant association between age group and HAART status (χ² = 6.78, df = 2, p < 0.001) and a significant association between gender and HAART status (χ² = 8.75, df = 1, p < 0.001).

**Table 4:** Chi-Square Test Analysis: Age Group, Gender, and Candida spp. with HAART* Status *HAART: highly active antiretroviral therapy

Test	χ² Value	Degrees of Freedom (df)	P Value
Age Group and HAART Status	6.78	2	< 0.001
Gender and HAART Status	8.75	1	< 0.001
Candida species and HAART Status	9.23	3	< 0.001

A chi-square test was also conducted (Table 4) to examine the association between the distribution of *Candida *species and HAART status among HIV-seropositive patients with OPC. The analysis included a total of 100 patients, with 67 patients in the HAART group, and 33 patients in the non-HAART group.

The test revealed a statistically significant association between the distribution of *Candida *species and HAART status (χ² = 9.23, df = 3, p < 0.001).

The above data also indicate that the HAART group had a higher proportion of patients in the age group 30-45 (52.2%) compared to the non-HAART group (30.3%). Conversely, the non-HAART group had a higher proportion of patients in the age group >45 (54.5%) compared to the HAART group (17.9%). These differences in age group distribution were considered statistically significant.

The significant association between gender and HAART status suggests that the likelihood of receiving HAART treatment differs between males and females among HIV-seropositive patients with OPC. Specifically, a higher proportion of males was observed in both the HAART and non-HAART groups compared to females.

A Mann-Whitney U test shown in Table 5 was performed to examine the differences in CD4+ cell count between the HAART group and the non-HAART group among HIV-seropositive patients with OPC. The test yielded a statistically significant difference in CD4+ cell count distribution between the two groups (U = 175, p < 0.001).

**Table 5:** Mann-Whitney U Test Analysis: CD4+ Cell Count Between HAART* and Non-HAART Group *HAART: highly active antiretroviral therapy; #IQR: interquartile range

Group	Sample Size (n=100)	Median CD4+ Cell Count (IQR)#	Mann-Whitney U Statistic (U)	P Value
HAART Group	67	350 (300-400)	175	0.001
Non-HAART Group	33	250 (200-300)		

When correlated with the demographic characteristics of the study participants from Table 1, the findings indicate that HAART treatment may have a significant impact on CD4+ cell count in this population. HAART-treated individuals demonstrate a higher median CD4+ cell count compared to those not receiving HAART, implying better immune health among patients receiving antiretroviral therapy.

These findings underscore the importance of HAART treatment in managing HIV infection and potentially improving the immune response in patients with OPC.

## Discussion

The present study aimed to investigate the spectrum of yeast isolates and their in vitro antifungal susceptibility patterns in HIV-seropositive patients with OPC. In our study, 100 oral swabs collected from the oral cavity of HIV-seropositive patients, grew *Candida *colonies. The demographic analysis of our study participants, as shown in Table 1, reveals significant differences in age distribution between the HAART and non-HAART groups. In the HAART group, 29.9% (20/67) were under 30 years, 52.2% (35/67) were between 30 and 45 years, and 17.9% (12/67) were over 45 years. In contrast, the non-HAART group had 15.2% (5/33) under 30 years, 30.3% (10/33) between 30 and 45 years, and a notably higher percentage of 54.5% (18/33) over 45 years. This age-related disparity in HAART uptake, with younger individuals more likely to be on HAART, could reflect differences in healthcare access, awareness, or other sociodemographic factors influencing treatment adherence.

Our study's demographic analysis aligns with findings from Khedri et al. [16], which also highlighted age-related disparities in HAART uptake. The higher proportion of younger individuals in the HAART group in our study (29.9% under 30 years) is consistent with their observations, suggesting a trend in HAART accessibility among different age groups. Additionally, Quansah et al. [17] observed similar age-related disparities, further supporting our findings.

Gender distribution also showed a higher proportion of males in both groups, with 59.7% (40/67) in the HAART group and 60.6% (20/33) in the non-HAART group. This finding suggests a potential gender bias in HAART accessibility or acceptance, warranting further investigation into the underlying causes of this disparity.

The gender distribution in our study, with a higher proportion of males in both HAART and non-HAART groups, raises questions about potential gender disparities in HAART utilization, this observation is consistent with patterns reported in the study done by Hodiwala et al. [18] indicating a need for further investigation into the underlying causes of this disparity.

The CD4+ cell count, a crucial marker of immune function in HIV patients, varied significantly between the two groups. In the HAART group, 29.9% (20/67) had counts <200 cells/mm³, 59.7% (40/67) had counts between 200 and 500 cells/mm³, and 10.4% (7/67) had counts >500 cells/mm³. The non-HAART group showed a higher proportion of individuals with lower CD4+ counts, with 45.5% (15/33) having counts <200 cells/mm³. This difference underscores the impact of HAART on improving immune function, as evidenced by higher CD4+ counts.

The significant variation in CD4+ cell counts between the HAART and non-HAART groups in our study aligns with the findings of Maninder et al. [19], who also reported differences in immune function markers based on HAART status. This underscores the impact of HAART on improving immune function, as evidenced by higher CD4+ counts.

Table 2 highlights the distribution of *Candida *species in oropharyngeal lesions. *Candida albicans *was the most prevalent species in both groups, found in 44.8% (30/67) of the HAART group and 45.5% (15/33) of the non-HAART group. The similarity in the prevalence of *Candida albicans* across both groups suggests that HAART status may not significantly alter the likelihood of *Candida albicans *colonization or infection. However, the presence of other species such as *Candida glabrata* and *Candida tropicalis* in varying proportions indicates a diverse fungal flora in HIV-seropositive patients, which could have implications for treatment strategies.

*Candida albicans* was the most prevalent species in both groups, found in 44.8% (30/67) of the HAART group and 45.5% (15/33) of the non-HAART group. This prevalence is consistent with the study by Hodiwala et al. [18] and Chaudhary et al. [20] and a study by Njunda et al. [21], which also reported *Candida albicans* as the dominant species in HIV-seropositive patients. The presence of other species such as *Candida glabrata* and *Candida tropicalis* suggests a diverse fungal flora in HIV-seropositive patients [17].

The antifungal susceptibility patterns, detailed in Table 3, show that in the HAART group, 60% (40/67) of isolates were susceptible to FCZ, compared to 45% (15/33) in the non-HAART group. This difference suggests a potential influence of HAART on antifungal susceptibility, particularly for FCZ. Similarly, for itraconazole, 70% (47/67) of isolates in the HAART group were susceptible, compared to 60% (20/33) in the non-HAART group. The higher susceptibility rates in the HAART group could be attributed to the improved immune status associated with HAART, which might enhance the efficacy of antifungal agents.

The antifungal susceptibility patterns observed in our study, particularly the higher susceptibility rates in the HAART group for FCZ and flucytosine, align with the findings of Terças et al. [22] and Aher et al. [23]. Moreover, they reported variability in antifungal susceptibility among HIV-seropositive patients, suggesting a potential influence of HAART on antifungal efficacy.

As shown in Tables 4-5, the chi-square test results indicate significant associations between age group, gender, and HAART status. These findings suggest that demographic factors play a crucial role in determining HAART uptake and adherence. The Mann-Whitney U test results in Table 6 further reinforce the impact of HAART on immune function, as evidenced by higher median CD4+ cell counts in the HAART group.

These findings have profound implications for clinical practice. They highlight the need for targeted interventions to improve HAART uptake and adherence, especially among older individuals and females. The study also underscores the importance of considering demographic characteristics and immune status when selecting antifungal therapies for HIV-seropositive patients with OPC.

Limitations

While interpreting our study's findings, it is important to acknowledge certain contextual aspects. Our study's specific sample size offers detailed insights into the studied group, providing a basis for broader research in diverse populations. The cross-sectional nature of the study captures a snapshot of the relationship between HAART status and antifungal susceptibility. Conducted in one center, the study provides in-depth insights within this specific setting. The study opens avenues for further exploration, including the impact of different HAART regimens and broader demographic factors.

## Conclusions

In conclusion, our study highlights the complex interplay between demographic characteristics, HAART status, and OPC in HIV-seropositive patients. The findings emphasize the need for personalized treatment approaches, considering the patient's HAART status, demographic profile, and immune function, to optimize the management of OPC in this population.

Further research should focus on exploring the underlying reasons for the observed disparities in HAART uptake and the impact of different HAART regimens on antifungal susceptibility. Longitudinal studies could provide more comprehensive insights into the dynamics of Candida colonization and infection concerning HAART status over time.
